# A Theoretical Discussion of How Common Understanding and Reflection Upon Need for Resources Can Prevent Risks Underlying Social Innovations

**DOI:** 10.1177/00469580231152078

**Published:** 2023-01-30

**Authors:** Hege Gjertsen

**Affiliations:** 1 UiT The Arctic University of Norway, Harstad, Norway

**Keywords:** social innovation, collaborative innovation, welfare services, wicked problems

## Abstract

The welfare state is facing complex challenges. Social innovation is considered as the solution to social challenges and so-called wicked problems, problems in the welfare state that are important, but difficult to solve. This implies being willing to take the risk that is involved when being innovative. A discussion of how different kinds of social innovations carry various risks and how some of these can be prevented, is still limited. This article looks at experiences from previous social innovations and ask what we can learn from them. It elaborates on why social innovation is challenging and what we can do to reduce the risk of failure. The research question is: *What risks are at stake in different social innovations, and how can these be prevented?* The article highlights risks and issues associated with social or collaborative innovation related to welfare services. It is theoretical and based on innovation theory and previous research, with examples from Norwegian welfare services. The purpose is to explore challenges and risks involved in 4 stereotype versions of social innovation as a result of crossing 2 dimensions of social innovation described above: (a) the degree of novelty and (b) who has initiated the innovation. The article enlightens some aspects of the implementation phase that are important to be aware of if we want to minimize the risk of failure. This concerns the importance of creating a common understanding of the innovation and reflecting on the need for extra resources.

## Introduction

The welfare state is facing complex challenges, which are unmet.^1,2^ Due to an ageing population and rising expectations for the content and quality of welfare services, municipal health and care services are facing both increased and new demands. Bommert^3^ claims that public sector innovation would not alone be able to solve today’s major challenges such as an aging society and climate change. Others stress that the public sector needs to find radically new ways of innovating.^4-6^ Collaborative practices and social innovations have been proposed to meet these challenges.^7,8^ Torfing^9^ stress the importance of social innovation: “The strength of collaborative innovation is that the interaction between public and private actors sharpens the problem definition, generate more and better ideas and build ownership to new and bold solutions” (p. 27).

Over the last decades, the focus on public, social, and welfare innovation has increased in Norway. Several public documents and research emphasize social innovation as the solution to social challenges and so-called wicked problems, problems in the welfare state that are important, but difficult to solve.^7,10-13^ In accordance with this, the government stresses the importance of the public sector and municipalities to be innovative when it comes to different services, both regarding the content and the organization of these. This implies being willing to take the risk that is involved when being innovative.^10,14^ According to Hartley^15^ innovation is uncertain in both process and outcome. The argumentation regarding the importance of innovation has stressed the risks and the importance of the municipalities’ willingness to move ahead, despite those risks.^16^ A discussion of how different kinds of social innovations carry various risks and how some of these can be prevented, is still limited.^17^ However, it is important to take a closer look at this to prevent, as far as possible, the failure of innovations. It may be a good idea to look at experiences from previous social innovations and ask what we can learn from them.^18^

This article elaborates on why social innovation is challenging and what we can do to reduce the risk of failure. The research question is: *What risks are at stake in different social innovations, and how can these be prevented?* The article highlights risks and issues associated with social innovation related to welfare services. It is not possible to provide specific recipes on “how to do” social innovation with a guarantee of success. Nevertheless, by examining different kinds of social innovations, the article will hopefully contribute to support innovation processes, both within public sectors and across public and voluntary or private sectors. By crossing 2 dimensions, the degree of novelty and where the innovation has been initiated, and presenting 4 stereotype versions of social innovation, we can learn something about risks and how to prevent these. The article aims to bring novelties to the research field by enlighten lessons learned from previous social innovations, within an analytical framework of 2 aspects of social innovation—the need for a common understanding of innovation and the resources that may be needed to implement the innovation.

Welfare services in Norway are mainly public and created mainly locally in the 356 (current) municipalities. Nevertheless, in some cases, welfare services are financed and/or organized by private companies or voluntary organizations. I will, therefore, refer to welfare services in Norway instead of talking about the public welfare sector. It is still a mainly public regulated context to which all welfare services must relate, and these laws and regulations are a context that differs from the context of private and competitive businesses. However, less focus has been on social innovation, including collaboration with private and voluntary sectors when it comes to welfare services.

The article is theoretical and based on innovation theory and previous research, with examples from Norwegian welfare services. It presents a descriptive analysis. The purpose is to explore and enlighten challenges and risks involved in 4 stereotype versions of social innovation. The article is structured as follows. First, I introduce the theoretical background for the discussion. Second, I present 4 stereotype versions of social innovations related to welfare services in Norway. Finally, I discuss the research question based on the 4 examples described.

## Theory of Social and Collaborative Innovation

Several quite similar concepts have been presented to capture innovation focusing on welfare services. While “public innovation” mainly refers to, or are associated with, innovation within the public sector, “social innovation” widens up, including the private and voluntary sectors as well.^19,20^ In this article I will use the concept of social or collaborative innovation, although some of the innovations I discuss just as well can be described as public innovations.

*Social innovation* is social in a double sense. It offers a new solution to a social challenge, and the solution involves several actors. In other words, both the reason and the solution are social. One definition of social innovations is that they are new solutions (products, services, and organizational forms) to social challenges (more than other alternatives) that at the same time create new sorts of collaboration.^21^ A general definition of social innovation is that it can be understood as the process or the result of using new knowledge, either by putting existing knowledge together in a new way or by using it in new contexts.^22^ It is about creating a positive social change, improving social relations, and collaborating to meet social needs. Social innovation includes a break with something. It does not necessarily have to be something radically new; it can, for example, be a copy, a so-called second mover, where we transfer something into a new context. We can study different aspects of social innovations. I have chosen to explore 2 aspects as an analytic framework, because they encompass important aspects of an innovation. This is firstly, the degree of novelty,^23^ and secondly, where it has been initiated.^21^

Social innovation can be placed on a continuum from radical on the one hand, and incremental on the other.^14,20^ More rapid and major changes characterize *radical innovations. Incremental innovations* are often described as step-by-step processes but still involve a break with previous practice. We can also talk about *everyday innovations* when employees find new ways to solve everyday challenges without it necessarily being defined as innovation.^24^ The sum of several incremental innovations can also result in a radical change over time. It is apparently easy to define the degree of novelty of an innovation, but sometimes that is not the case. An innovation defined as incremental, and therefore assumed quite unproblematic to implement, can be considered as far more radical from a different point of view. For example, leaders in an organization can consider it easy to implement a new work task or organization, while frontline service providers can consider it to involve a major change in their working day or in the “workplace culture.”

Social innovation can further be defined by who initiated it. Where did it come from, from those supposed to implement it, or outside the organization? Another relevant question related to who initiated the innovation, is whether the solution is an answer to a challenge experienced at “the floor”, by those working at the frontline in the welfare services. We can talk about top-down (management-driven) and bottom-up (user- or employee-driven) innovations.^21^ Social innovations can be defined anywhere along this continuum.

One form of social innovation is *collaborative innovation*, which is about several actors collaborating to be innovative. Nambisan^6^ defines collaborative innovation as a “collaborative approach to innovation and problem-solving in the public sector that relies on harnessing the resources and the creativity of external networks and communities (including citizen networks as well as networks of non-profits and private corporations) to amplify or enhance the innovation speed as well as the range and quality of innovation outcomes” (p. 11). Being successful with collaborative innovation is largely about finding someone to get along with. Collaborative innovation opens the innovation cycle to a variety of actors and taps into innovation resources across borders. In other words, the innovation process is opened; actors from within the organization, other organizations, the private and third sector, and citizens can be integrated into the innovation cycle (idea generation, selection, implementation, and diffusion) from the earliest stage onwards. Kobro^21^ stress that “good collaborative social innovation work is characterized by the fact that people with different resources, experience and knowledge work together” (p. 4) Andersen et al^25^ underline that communication is an essential aspect of collaborative innovation, to prevent difficulties and as a tool if challenges occur.

Collaborative innovation is expanding the focus to include collaboration between public sectors, or between private and public sector.^7^ However, it is still innovations related to welfare services and, therefore, associated within the context of the public sector in Norway. The fact that those supposed to collaborate in many ways can be considered living on different “planets” regarding rules and values, has to a limited extent been discussed or reflected upon. Collaborative innovations involving different contexts can be challenging. Public innovations are supposed to meet other purposes than innovations in competitive businesses in the private and public sectors, and they are also subject to other laws and regulations. While innovation in the private sector is linked to increased profit and developing better solutions, social innovation also focuses on increased quality of welfare services, and increased efficiency (often related to the municipalities’ budgets). The concept of value in the public sector is somehow challenging. We can distinguish between value for the municipality and value for the service receivers.

The theory of social innovation often stresses risks that is always involved.^3,9,26^ It is an aspect of uncertainty involved in every innovation as opposed to ordinary development and changes in an organization.^1,27-29^ According to Brown and Osborn,^30^ most prior conceptualizations of risk usually include an element of probability that an event might happen and that there will be gains and losses associated with it. Therefore, they relate the concept to uncertainty.

While risk regarding innovation in the private sector mainly concerns economic aspects, risk regarding innovation in the public sector also concerns the quality and extent of the services affected. Risk is involved in all parts of the innovation process. Although the innovation process seldom is linear, we can discuss these phases: problem identification, choosing a solution, implementation, and diffusion.^7^ The implementation is the most crucial phase.^21,31^ That is where the idea becomes an innovation. Still, all the phases imply challenges and risks to be aware of. Furthermore, there is a common understanding of radical innovations being more risky than incremental. For example, Aasen and Amundsen^32^ emphasize that incremental innovations are supposed to be less risky than radical innovations.

Brown and Osborne^30^ stress that it is not possible to avoid all kinds of risks; we must cope with them throughout the innovation process. They argue that the management of risk in innovation has received less attention than it deserves and that discussions of the assessment of and coping with risk are rare in public services. Risk related to innovation should not be minimized but seen as an essential element to be governed in practice.

## Four Stereotype Versions of Social Innovation Related to Welfare Services

Below, I will examine 4 stereotypes of social innovations as a result of crossing 2 dimensions of social innovation described above: (a) the degree of novelty and (b) who has initiated the innovation. Both the degree of novelty and where it has been initiated play a crucial role regarding the risk of failure. Moreover, as discussed later in the article, implicitly different kinds of social innovation require the consideration of special precautions. By crossing the 2 dimensions of social innovation I will illuminate some new aspects of risks involved. The different combinations involve specific risks and require different solutions and considerations.

I want to start with presenting a classic cross (4-field) table, including the 2 dimensions of social innovation (Table 1). This results in 4 stereotype versions of social innovations. Using examples from Norway, I will describe all 4 combinations of these 2 dimensions, and elucidate the related challenges experienced. I will underline which actors are involved. I will not discuss how the innovations have been experienced by those receiving these welfare services. Neither will the 4 social innovation examples be evaluated or examined in detail. The purpose is to exemplify 4 stereotype versions of social innovations as a starting point for the discussion of risks related to social innovations later in the article.

**Table 1.** Four Stereotypes of Innovations With Examples From Norwegian Welfare Services.

	Radical	Incremental
Top-down	The NAV-reform	Work method for activating nursing home residents
Bottom-up	Incest centers	Everyday innovation in nursing homes

### The Norwegian Labor and Welfare Administration (NAV) Reform

The first version of innovation is characterized by being initiated top-down and involving major changes. The Norwegian Labor and Welfare Administration (NAV^I^), called the NAV-reform can illustrate this kind of innovation. NAV was formally established on July 1, 2006 and implemented stepwise over time from 2006 to 2011 at 457 NAV offices^33,34^ in municipalities and city boroughs.^II^ Some offices are large, with over 100 employees, while others (app. 1/3 of the offices) have fewer than 3 employees. The reform was adopted by a broad majority of the Norwegian Parliament. Until 2006, the Labor and Welfare Administration was fragmented, involving 3 large public agencies (employment and national insurance administration, and part of the social assistance services) with limited collaboration. The reform merged these government agencies into a new organization called NAV, after the “one door for all” principle (“one-stop shops”).^35^ The aim was to improve cooperation and coordination between different services.^34^ The goal of the reform was to (a) increase work participation and reduce people receiving welfare support (according to the “work line”), (b) create a more user-friendly system (users getting better help and increased influence), and (c) create a more efficient labor and welfare system.

Champion and Bonoli^36^ characterize the NAV-reform as “one of the most radical coordination initiatives adopted in Europe” (p. 325) The NAV-reform is also one of the largest public sector reforms in recent Norwegian history. This is because of the scope of the reform. NAV spends approximately 1/3 of the national budget, encompasses a very wide range of services and benefits, which implies that 50% of the population are in contact with NAV each year^37^ and because the reform involved a merger of 2 large state agencies followed by mandatory partnerships between the central government and each municipality. It implied a more formal collaboration between the new state administration and the local government social services administration. The new state agency entered partnerships with the municipalities and would work with employees of the former municipal social service offices. Many thousand employees were affected and had to change their workplace and/or work tasks.

The NAV-reform is mainly a structural reform involving organizational changes.^38^ The substance of the welfare services remained little changed in the implementation period, with some exceptions concerning for example a work program.^III^ Therefore, to some degree, we can also talk about a content reform.^39^

Research concerning the implementation of the NAV-reform has pointed to several challenges.^37^ These imply, among other things, difficulties uniting 3 different agencies with different practices, histories, and work tasks (and using different computer systems); implementing the reform resulted in significantly increased work pressures for the employees and a lack of competence needed to follow up with users.^34^ Although the decision to implement the reform was taken by the government, the local NAV offices could, to some degree, decide how to organize the new office. However, the government still had expectations, and the local NAV offices had little influence on important decisions^40^(p. 173).

### A New Work Method to Increase Activity at Nursing Homes

The second version of innovation is also initiated outside the organization that is supposed to implement it; in other words, a top-down innovation. However, this version includes apparently minor changes. I will illustrate this stereotype version of social innovation by examining a new work method to systemize work related to activities for residents in nursing homes in a Norwegian municipality. This is not a completely new work method, but it was new in these nursing homes and, therefore, a so-called “second mover” innovation.

In 2013 to 2015, a small municipality in northern Norway with a population of approximately 6000 implemented a project in some nursing homes to activate the residents.^41^ The idea of this new work method, and the decision to implement it, was not taken by those working at the nursing homes but by politicians and the administration in the municipality. The new work method was implemented as a project and then implemented as a part of ordinary practice at the nursing homes. Public reports stressing that old people living at nursing homes are often inactive and that the municipalities are responsible for doing something about it formed the background for the decision.^11,42^ The white paper “Future care”^42^ underlines the importance of more active care and increasing the users’ influence, giving nursing homes residents a better quality of life.

The goal of the new work method was to increase the systematic work when it comes to activating the nursing homes residents in the municipality and give the residents a more meaningful everyday life. This implied a change in the culture at the nursing homes from a care culture to an activity culture. Although the new idea and the decision were taken outside the organization that would implement them, the implementation had to be in close collaboration with nursing homes employees, the resident’s relatives, and collaboration partners such as voluntary organizations.

As mentioned, the employees at the nursing homes were not involved in developing the innovation. Still, they were included in the implementation process. It was presented as a new work method, not including much more work, but mainly a change “in focus” at the nursing homes. Those working at the nursing homes as health care workers or unskilled had another opinion. In an evaluation of the project, the employees stressed several factors as relevant for difficulties implementing the innovation; among other things was a lack of enough resources and how the innovation had been presented and rooted in the organization.^41^

### Support Centers for Victims of Incest and Sexual Abuse Established by Peers

The third version of innovation is characterized by being initiated from “the bottom,” involving those supposed to implement it, at the same time including a radical change when it comes to the welfare service they will offer. I will exemplify this kind of social innovation by looking at the implementation of the incest centers in Norway. There are currently 23 centers against incest and sexual abuse, at least 1 in each of the 19 counties.^43^ These centers offer important support for victims of incest and sexual assault, although they are not part of the public health system but are a supplement to governmental and municipal services. The centers may also function as a door-opener into other public services where the users are not already receiving such help.

The main services offered at these centers are individual and group conversations with professionals, in addition to, among other things, social activities such as meals, activities, courses, and groups, and providing information about sexual abuse in primary, secondary and upper secondary schools and occasionally also in kindergartens.^44^ The first incest centers in Norway were established in the 1980s. The majority were established in the 1980s and 1990s. They were based on a self-help-ideology and the principle of peers. People themselves, having experienced sexual abuse or the next of kin to a survivor of sexual abuse, wanted to help others, as a reaction to how victims were treated in mental health services: seen as a patient, diagnosed, and offered standard treatment. Initially, the centers were small and relied on voluntary work and private donations. Today, The Norwegian Directorate for Children, Youth, and Family Affairs (Bufdir) funds the centers in cooperation with municipalities, counties, and regional health authorities. However, the centers must still secure a small proportion of their funding from local sources, both private and public. In other words, the centers must first receive financial support from the municipalities, counties, and regional health authorities, and then this triggers funds from Bufdir.^43^

According to the users, the centers have been of tremendous importance, offering help that they cannot receive anywhere else.^44^ These centers have traditionally operated at the grassroots level and outside the statutory system of services for victims and with an open-door policy for all victims. They have, however, undergone a profound transformation over the last years. They are no longer staffed by volunteers but by a mix of paid social professionals.^43^ In this article, I focus on the implementation of the centers and the first decades afterward.

The incest centers were intended to be a supplement to the public help system, but they offered a help no one else could give this group, so we can talk about a new kind of help. Moreover, where these centers were established, it was a major break from previous support offered. The establishment of the incest centers can therefore be defined as a radical innovation initiated from “the bottom.”

Evaluations of the incest centers in Norway point at several challenges.^44,43^ The centers’ finance was challenging for many years and still is. Other challenging aspects are related to what kind of competence is needed to work at the centers. Is being a survivor of incest sufficient, or is formal education necessary? Further, the extent and the quality of cooperation between different municipal and regional services^IV^ and the incest centers vary. Some of the cooperation that occurs will be of an ad hoc character, often relating to specific cases. In other cases, incest centers are participating in interdisciplinary teams of cooperation, for instance, consulting teams.

### Everyday Innovation in a Nursing Home: Yellow Notes for Information Exchange

The fourth version of innovation is also characterized by being initiated from the bottom but is not expected to involve major changes. As I will stress in the discussion, and this may be obvious, this kind of social innovation involves the least risks. I will illustrate this by describing a new way of organizing information exchange at a nursing home. As a student project part-time master’s students working in different welfare services, had to develop and implement a small innovation project at their workplace. One of the students, working at a nursing home, experienced challenges regarding information delivering from one employee shift to another. Due to time pressure, they often did not find time to tell everything of importance regarding the residents to the next shift, and those employees starting to work did not have time to read everything written in the patient journal. The student discussed this challenge with co-workers, and they came up with a new solution to this problem. The innovation involved writing yellow papers with information in short text and attaching them to a door where everyone at work could read them. This was a new solution to a problem at this workplace. It was new (in this context), experienced as useful, and implemented. The employees got permission to do this after asking the department leader. Moreover, the implementation of the new practice did not trigger the need for additional resources.

One challenge regarding this kind of innovation is the possibility of not being recognized as innovation. Lippke and Wegener^24^ are afraid that everyday innovations often are invisible and that we can ignore important aspects of innovation where actors find creative solutions, improvise, and adjust related to actual conditions in their everyday work. Further, Wegener and Tandgaard^45^ also stress that everyday innovations require that people have access to working creatively, and it also requires service providers to have problem-solving abilities.

## Discussion: Risk of Failure Regarding Social Innovations and How to Prevent It

When you have come up with a good idea, in this case, related to welfare services, whether it is a new product, service, or organization, the next question is; how can you implement it? Whether or not the innovation ends up as a success depends on a variety of conditions and implies the whole innovation process. However, in the discussion below, I will answer the research question in the article by focusing on the implementation phase. Several factors are important or necessary to make the implementation a success.^7,46^ In the discussion I will emphasize some important aspects concerning (a) a common understanding of the innovation and (b) resources needed to implement the innovation idea. Both theory^5,19^ and research^41^ stress these as important aspects regarding implementation of innovations. As briefly described above, all 4 stereotype versions of social innovations related to welfare services include challenges, although not all in the same way and degree, and these can be met in different ways.

### A Common Understanding of the Innovation

First, it is necessary that those implementing the innovation and those who initiated it have a *common understanding* of the purpose of the innovation, the content of the innovation, how it best can be implemented, and what challenge it is intended to resolve. Important keywords are collaboration, communication, and anchoring.

One way to ensure a common understanding of the innovation is collaboration right from the start. If the innovation is developed “bottom-up,” this is often not a problem since those experiencing the problem are the same people who come up with the solution and are supposed to implement it. Still, this may be challenging. Important questions to ask: Are everyone (or every occupational group) affected at the workplace involved in identifying the problem and discussing solutions? Moreover, are those affected by the innovation, especially service receivers, involved in any way? When we are talking about everyday innovations, incremental, and bottom-up innovations, it may also be necessary to inform or get permission from the leader.

Collaboration from the earliest stage onwards is valuable for several reasons. Challenges concerning collaborating during the innovation process are often more present at top-down innovations. If we are not talking about bottom-up innovations, a common understanding also lays the foundation for joint ownership of the innovation. Kobro^21^ stress the importance of anchoring: “It is precisely because the activity is about change, that there is often greater room for uncertainty. Secure anchoring is therefore important” (p. 34). The further away the identification of the problem is, and the development of and decision to implement the innovation is taken, the more likely it is that those supposed to implement the innovation have not been involved. The NAV-reform is a good example of this. The service providers at the NAV offices were not involved in developing the reform. According to Brown,^26^ there is a gap between the government’s focus on innovation in the public sector and service providers’ ability to implement them.

This brings us further to another way to ensure a common understanding of the innovation. When the innovation is developed elsewhere, and the decision to implement it is taken “top-down,” it is crucial to focus on the presentation of the innovation. One challenge when it comes to top-down innovations is the need to communicate the importance of the new idea and establish a feeling of ownership at “the floor” among those who are going to implement the innovation. When and how is it presented to those who are going to implement it, often service providers? What arguments are used to “convince” them, and what “language” is used? When the municipality presented above tried to implement the new work method for the activation of nursing homes residents, they failed by using a far too academic language, that unskilled service providers did not understand, or at least it created a distance or diversity between those developing the innovation and those implementing it.^41^ Incremental top-down innovations may seem unproblematic to implement. However, even though the new practice does not imply a major break with the status quo, it can be difficult to implement if the service providers do not understand the meaning of the wanted change or do not think it is a good idea. So, when implementing a top-down innovation, communication is crucial, even if we are talking about quite minor changes.

Communication is also very important when implementing innovations when several actors are involved across sectors or across public, private, and voluntary sectors. In principle, actors can manipulate the elements of the innovation cycle to exert their interests over the goal of innovating public value.^47^ Sørensen and Torfing^7^ underline that “. . . efforts must be undertaken in order to reduce the risk of conflicts between the many different stakeholders” (p. 3). According to Fung,^48^ actors from the private sector participating in collaborative innovation can “hijack” the decision-making process. Sifry^47^and Fung^48^ describe this problem because of deliberate manipulation or hidden agenda. Moore and Hartley,^49^ on the other hand, stress that collaboration with various actors entails a transfer of “decision rights.” They point out that if different actors invest their resources into the collaboration, they claim to have a say in the production of public value. Bommert^3^ points out that: “Accordingly, actors contributing with their innovation resources to the innovation cycle will demand a right to determine at least to an extent what idea of public value is generated, selected, implemented, and diffused. Especially if one considers the case that the government plays only a minor or no role in collaborative innovation, it gives up partly or entirely its authority of defining public value” (p. 26). This may be the case for the incest centers in Norway. In the beginning, the peers established the centers and made all decisions. The government contributed only with economic support. Over the years, 2 major changes have occurred when it comes to the organization of the centers: They have increased their collaboration with public welfare services, and the staff now have health and social care education, and more seldom, their own experiences as sex abuse victims. There have been discussions about what kind of services the centers should provide and their role in similar public services.^44^

### Are Extra Resources Needed?

Second, it may be necessary with *extra resources* to implement social innovations, often depending on the degree of novelty. This may be about having enough time to do extra work tasks or buy equipment. It often requires more people and/or money to implement radical innovations than incremental, especially if it involves major changes in the organization or content of the welfare service. The NAV-reform illustrates this. It was not sufficiently reflected upon. Work pressure is an ongoing challenge at many NAV offices.^37^

In the case of bottom-up innovations, 1 challenge is to convince the leaders or government of the necessity of the innovation, to get permission to implement the new idea, involving changes in the organization or the content of welfare service, or to get funding or permission to spend time on implementing the innovation. As discussed above, bottom-up innovations like the support centers for victims of incest and sexual abuse need to convince the government about their importance in receiving money. It is not a statutory service, and they have struggled for years to convince the government about the importance of receiving economic support.^43,44^ This shows that even though bottom-up innovations may seem easier to implement, they still may be challenging since the new idea or practice is anchored at the implementation level. Will the new idea demand more resources like money or more people? Does it demand more or another competence? Especially if we talk about radical innovation, it must be anchored at all levels. If it is an incremental innovation, which does not need more resources, this may not be so important. One example that may prove that this is wrong is the implementation of a new work method for activating nursing home residents. The government in the municipality and the leaders were sure at the beginning that the implementation would be unproblematic since it only involved some minor changes concerning the work tasks at the nursing homes. As the evaluation^41^ pointed out, those going to implement the new work method disagreed. They called for more resources and emphasized the extra work tasks the innovation implied. Among other things, the employees had to document everything, and there were expectations of participating in several meetings, also when they were off work, without getting paid.^41^

## Conclusion, Limitations of the Study, and Some Research and Policy Implications

This article discusses essential questions concerning risks of social innovation related to welfare services in Norway. By looking at 4 stereotype versions of social innovation, I have enlightened some aspects of the implementation phase that are important to be aware of if we want to minimize the risk of failure. This concerns the importance of creating a common understanding of the innovation and reflecting on the need for extra resources. All 4 combinations of the 2 dimensions, “degree of novelty” and “top-down versus bottom-up,” imply different risks to be aware of. Still, the version most challenging is the combination of top-down and radical innovation since it requires the largest break with today’s practice and is initiated outside the organization implementing the innovation. Not surprisingly, the fourth version of innovation described, bottom-up, and incremental, has most chance to succeed. However, as discussed in this article, all kinds of innovation involve risk, which implies precautions be taken. This challenges decisionmakers when initiating social innovations. Hopefully, the article can contribute to better designed policies.

Limitations in the analysis in the article are related to delimitations I made. I cultivated 4 stereotype versions of social innovation to highlight risks involved and how these can be prevented. The reality is, of course, much more complex. By including other dimensions of social innovation, the discussion could have been extended.

## Notes

I. NAV is the short name for the Norwegian Labor and Welfare Administration. NAV was originally short for the New Labor and Welfare Administration. NAV is now a standalone name and a brand.

II. In 2006, 25 pilot NAV offices were established, 121 in 2007, 147 in 2008, 154 in 2009, 9 in 2010, and 1 in 2011.^50^

III. The qualification program (2007) and work assessment allowance (AAP) (2010).

IV. Collaboration with other, municipal offices such as the public health nurse, the family advisory office, the child welfare council, the police, NAV, and GP physicians, are important for the incest centers. Regional offices important to collaborate with are the RVTS (regional resources center on violence, traumatic stress, and the prevention of suicide), the DPS (Community Mental Health Center), the emergency ward, childcare centers, BUP (psychiatric services for children and young people), and addiction treatment centers.
